# Association between Phytate Intake and C-Reactive Protein Concentration among People with Overweight or Obesity: A Cross-Sectional Study Using NHANES 2009/2010

**DOI:** 10.3390/ijerph16091549

**Published:** 2019-05-02

**Authors:** Seth M. Armah

**Affiliations:** Department of Nutrition, University of North Carolina at Greensboro, Greensboro, NC 27412, USA; s_armah@uncg.edu

**Keywords:** phytate, C-reactive protein, inflammation, body mass index

## Abstract

Phytic acid has anti-oxidant properties, which are useful in addressing inflammation. This study investigated the relationship between dietary phytate intake and C-reactive protein (CRP) levels among individuals that are overweight or obese. The study used cross-sectional data from the 2009/2010 National Health and Nutrition Examination Survey (NHANES) for 3152 subjects. Phytate intake was estimated using phytate content of foods reported by the International Zinc Nutrition Consultative Group (IZiNCG). Logistic regression was used to determine the associations between phytate intake and odds of elevated CRP concentration (CRP >3 mg/L), adjusting for confounders. Medians (and 95% CIs) for phytate intake and CRP concentration were 0.66 (0.64, 0.68) g/d and 1.4 (1.2, 1.5) mg/L, respectively. Phytate intake was higher in males than females, higher in non-Hispanic Whites than non-Hispanic Blacks and Mexican Americans, and lower in current smokers than former smokers and nonsmokers. Higher phytate intake was associated with lower odds of elevated CRP (OR = 0.66; 95% CI = 0.52, 0.84). Women, as well as current and former smokers with overweight or obesity, had higher odds of elevated CRP concentration. These results imply that individuals with high phytate intake, particularly among those with overweight or obesity, have lower risk for inflammation-related chronic diseases such as cardiovascular diseases.

## 1. Introduction

Phytic acid, also known as inositol hexakisphosphate or IP6, is found in grains, oil seeds, legumes and nuts as the primary storage form of phosphorus, storing as much as 80% of phosphorus [1]. In its salt form, it is referred to as phytate. Phytic acid is a well-known inhibitor of zinc and iron absorption [2,3,4,5]. For this reason, its consumption is a concern, particularly in developing countries where these micronutrient deficiencies are rife. Despite its adverse effect on mineral absorption, phytic acid is also a potent anti-oxidant implying strong health benefits. In animal and cell culture studies, it was shown to have anti-neoplastic properties in various types of cancer including breast, colon, liver and prostate cancer, as well as anti-inflammatory properties [6,7,8,9,10]. The health benefits of phytic acid are largely attributed to its anti-oxidant properties. Phytic acid has a strong affinity for iron and can chelate free iron, thus preventing iron-related free radical formation and oxidative damage [11]. As part of the human body’s normal cellular metabolism, free radicals are generated and are important for the activation of various metabolic pathways. However, a healthy balance between free radical generation and the availability of anti-oxidants is necessary to prevent oxidative stress, which can lead to various adverse health outcomes including diabetes, cancer, cardiovascular diseases (CVDs) and inflammation [12]. Dietary anti-oxidants are important in maintaining this balance and thus preventing inflammation. C-reactive protein (CRP) is a well-studied, non-specific inflammation marker that is synthesized mainly in hepatocytes. CRP is elevated in various health conditions such as obesity and insulin resistance, and higher levels are also associated with an increased risk of developing CVDs and diabetes [13,14,15,16,17,18]. Despite the numerous studies in animals and cell culture on the health benefits of phytic acid, there is a dearth of information on the association of dietary phytic acid intake and markers of inflammation in a population-level study. This study focuses on individuals with overweight and obesity because obesity is associated with low grade chronic inflammation marked by increased production of adipocyte cytokines, leading to an increase in hepatic CRP production [19,20,21,22]. The aim of the study was to investigate the association between phytate intake and CRP concentration among subjects 20 years and older that were classified as overweight or obese in the National Health and Nutrition Examination Survey (NHANES), using the 2009/2010 survey cycle. Due to its anti-oxidant properties, I hypothesized that phytate intake would be associated with lower odds of elevated CRP concentration among the study population. 

## 2. Materials and Methods

### 2.1. Data Source

Data for this study were obtained from the 2009/2010 survey cycle of NHANES [23] and the Food Patterns Equivalents Database (FPED) [24]. The NHANES is a cross-sectional survey that is conducted by the National Center for Health Statistics of the United States, a branch of the Centers for Disease Control and Prevention (CDC). The survey uses complex multistage sampling to assesses the health and nutritional status of non-institutionalized adults and children in the United States with oversampling for certain population subgroups. The survey was conducted according to the guidelines laid down in the Declaration of Helsinki and all procedures involving human subjects/patients were approved by the National Center for Health Statistics (NCHS) research ethics review board (Protocol #2005-06). Written informed consent was obtained from all subjects. The FPED database, on the other hand, converts foods that are reported in the NHANES study into the USDA Food Pattern Components such as fruits, vegetables, grains, dairy, protein foods, added sugars, oils, solid fats, alcoholic drinks and their subcomponents [24]. From the NHANES dataset, readily-available data for this study include CRP concentration, body mass index (BMI), demography, medical history, dietary fiber intake, female hormone use, and smoking data. CRP concentration in the NHANES survey was measured by latex-enhanced nephelometry using a Behring Nephelometer. Anti-CRP antibodies were covalently linked using particles made up of a polystyrene core and a hydrophilic shell. Test samples were diluted and mixed with latex particles coated with mouse monoclonal anti-CRP and the CRP in the samples formed an antigen antibody complex with the latex. CRP concentrations were calculated using a storable logit-log function calibration curve after automatic blank subtraction. Phytate intake data was not available from the NHANES survey data and was therefore estimated as previously described [25]. Briefly, the phytate content of food groups were obtained from a publication by the International Zinc Nutrition Consultative Group (IZiNCG) [2]. This was merged with the appropriate FPED data for the 2009/2010 survey cycle of What We Eat in America (WWEA) to estimate the phytate content of each food item based on the amount of the different food groups present. The different food groups used for this estimation were dark green vegetables, potatoes, other starchy vegetables, beans and peas, whole grains, refined grains, soy products, and nuts and seeds. In this study, phytate was used as a generic term to refer to both phytic acid and its salt.

### 2.2. Inclusion and Exclusion Criteria and Subject Categorization

Subjects included in this study were those 20 years and older with a BMI of ≥25.0 kg/m^2^ [26], and who had measurement for CRP concentration and data for estimation of phytate intake were available. Subjects with CRP of >10 mg/L and a white blood cell count of 10,000 cells/µL were excluded, as those levels could indicate an acute infection. Pregnant women were also excluded from this analysis. Out of 9754 subjects, a total of 3152 subjects met the inclusion criteria and thus qualified for inclusion. Apart from phytate intake and CRP concentration, other variables namely fiber intake, female hormone use, smoking status and medical condition status were included in the analysis because these variables were shown to influence CRP concentration. Subjects were put into one of three smoking categories namely never, former or current smokers [27]. This classification was based on two questions: “Smoked at least 100 cigarettes in life?” and “Do you now smoke cigarettes every day, some days, or not at all?”. The never smokers were those who responded never to smoking 100 cigarettes in life. Current smokers were those who reported smoking at least 100 cigarettes in life and who now smoke. Former smokers were those who reported to have smoked at least 100 cigarettes in life but currently do not smoke. Subjects were put into two categories for medical condition. Individuals with a history of at least one of the following conditions were considered to have a history of a medical condition related to inflammation: Diabetes, cancer, arthritis, and cardiovascular diseases (congestive heart failure, coronary heart diseases, angina/angina pectoris, heart attack, and stroke). Female hormone or hormone replacement therapy (HRT) use was also put into three categories, namely current, former, and never users as described by Crespo et al. [28]. Regarding inflammation status, subjects were classified as having a normal (≤3 mg/L) or an elevated (>3 mg/L) CRP concentration. 

### 2.3. Data Analyses

Data were analyzed using R Software Version 3.2.0 [29]. The package “Survey” of the R Software was used for statistical analysis taking into consideration the appropriate sampling weight, strata, and primary sampling unit (PSU). Medians and their 95% CIs were reported for phytate intake and CRP concentration since both datasets were skewed. The primary outcome variable for this study was CRP concentration, and the independent variables were phytate intake, sex, age, ethnicity, smoking status, HRT status, fiber intake and medical condition status. The results for only three ethnicity groups (non-Hispanic Whites, non-Hispanic Blacks and Mexican Americans) were presented. This was because of the small sample sizes and the heterogeneity for other races and other Hispanic groups. Mood’s median test was used to compare median phytate intakes and CRP concentrations among different categories and Bonferroni corrections for multiple comparisons was applied where more than two comparisons were made. Logistic regression was used to determine the associations between the independent variables and the odds of an elevated CRP concentration. The odds ratios (ORs) and their 95% confidence intervals were reported. Statistical significance was set at *p* ≤ 0.05. In the comparison of medians where Bonferroni corrections for three comparisons were applied (smoking status, ethnicity and HRT use), a *p* ≤ 0.0167 was used to determine statistical significance.

## 3. Results

After adjusting for sampling weight, strata, and PSU, 45% of the subjects were female and 55% were male, as seen in Table 1. Most of the subjects (68%) were non-Hispanic Whites, with 10% and 11% being Mexican Americans and non-Hispanic Blacks, respectively. Among the study subjects, 57% never smoked, 15% were current smokers and the remaining 28% were former smokers.

The median (with 95% CIs) phytate intake for all subjects was 0.66 (0.64, 0.68) g/d, as seen in Table 2. Median phytate intake was significantly higher among men than among women (*p* < 0.0001). Among the different ethnicity groups, non-Hispanic Whites had significantly higher phytate intake than both non-Hispanic Blacks and Mexican Americans (*p* < 0.0167 for both). Current smokers had lower phytate intake than both non-smokers and former smokers (*p* < 0.0167). The median (with 95% CIs) CRP concentration for all subjects was 1.4 (1.2, 1.5) mg/L. It was higher among women compared to men (*p* = 0.001), but did not differ by ethnicity or smoking status. Individuals with a history of at least one of the medical conditions identified in the methods section had higher CRP concentrations than those with no history (*p* = 0.0001) and former users of any kind of HRT had a significantly higher median CRP than those who have never used HRT (*p* < 0.0001).

When subjects were classified as having a normal or elevated CRP concentration (CRP >3 mg/L), CRP was elevated in 32% of subjects. Elevated CRP concentrations were observed in 42% of female subjects compared to 23% of males (OR = 2.4; 95% CI = 2.0, 2.9; *p* < 0.0001; see Table 3). Non-Hispanic blacks had higher odds of elevated CRP compared to non-Hispanic Whites (OR = 1.58; 95% CI = 1.28, 1.96; *p* = 0.001). Current smokers also had higher odds of elevated CRP compared to those who have never smoked (OR = 1.27; 95% CI = 1.04, 1.55; *p* = 0.03), and former HRT users had significantly higher odds of elevated CRP compared to those who never used HRT (OR = 2.09; 95% CI = 1.59, 2.76; *p* < 0.0001). Both phytate intake (OR = 0.69; 95% CI = 0.59, 0.81; *p* = 0.0005) and fiber intake (OR = 0.98; 95% CI = 0.97, 0.98; *p* < 0.0001) were associated with reduced odds of elevated CRP concentration in their respective logistic regression equations.

In the multiple logistic regression model (Table 4), phytate intake, sex and smoking status were significantly associated with the odds of elevated CRP. Higher phytate intake was associated with lower odds of elevated CRP concentrations (OR = 0.66, 95% CI = 0.52, 0.84; *p* = 0.04) and women had higher odds of elevated CRP compared to men (OR = 2.20; 95% CI = 1.84, 2.63; *p* = 0.003). Both current smokers (OR = 1.35; 95% CI = 1.12, 1.62; *p* = 0.05) and former smokers (OR = 1.40; 95% CI = 1.16, 1.69; *p* = 0.04) had higher odds of elevated CRP concentration compared to those that never smoked.

## 4. Discussion

Despite its inhibitory effect on mineral absorption, phytate possess potent anti-oxidant properties which gives it important therapeutic value. Phytate was shown to be anti-neoplastic and anti-inflammatory in animal and cell culture studies [6,7,8,9,10]. This study investigated the relationship between phytate intake and the odds of elevated CRP concentration among participants 20 years and older with overweight or obesity in the NHANES 2009/2010 survey cycle. The median daily phytate intake among subjects was 0.66 g. This is higher than a previously reported value of 0.60 g [25], primarily due to differences in inclusion/exclusion criteria for the different studies. While the present studies included only adults with overweight or obesity, the previous one included both children and adults and did not exclude subjects with underweight or normal weight status. Phytate intake was higher in men than women, lower in non-Hispanic Blacks and lower in smokers than non-smokers. These findings are consistent with previous studies. For example, Maclean et al. [30] reported poorer diet quality among smokers compared to nonsmokers and Nielsen et al. [31] reported higher intake of nuts, a rich source of phytate, among non-Hispanic Whites than both non-Hispanic Blacks and Mexican Americans.

The results of this study indicate that among individuals with overweight or obesity, higher phytate intakes is associated with lower odds of elevated CRP concentration. In obesity, the adipose tissue produces adipokines that induce the production of reactive oxygen species (ROS). The ROS lead to oxidative stress through mechanisms such as fatty acid oxidation [32], and trigger inflammation. CRP is a non-specific marker of inflammation produced by the hepatocytes in the liver, and its concentration is increased in obesity. Phytic acid may influence the inflammatory process through its anti-oxidant properties. The antioxidant properties of phytic acid are based on its ability to prevent iron-mediated free radical formation, and the suppression of lipid peroxidation [33,34]. Da Silva et al. [35] found that phytic acid treatment in jejunal explants of pigs exposed to fumonisin B1 and deoxynivalenol decreased thiobarbituric acid reactive substances levels and cyclooxygenase 2 expression, and increased levels of the reduced form of glutathione, implying an improvement in oxidative stress markers. In addition, Liu et al. [36] also showed that phytic acid treatment decreased serum concentration of TNFα, interleukin 6 (IL-6) and IL-1β in male Wistar rats. All these studies support the anti-oxidant/anti-inflammatory function of phytic acid. The findings of this study highlight the importance of dietary phytate intake in reducing the risk of various chronic diseases that are related to CRP concentration such as CVDs and diabetes. CRP has been demonstrated to be predictive of cardiovascular event risk through its interaction with low density lipoprotein and very low density lipoprotein [18]. Hu et al. have also suggested that CRP may mediate the relationship among other inflammatory markers such as IL-6, and type 2 diabetes [17]. 

Apart from phytate intake, sex and smoking status were found to be associated with the odds of elevated CRP concentration using logistic regression. While the association observed between phytate and CRP is novel, smoking and sex are well-known predictors of CRP concentration. Cigarette smoking results in exposure to reactive oxidant substances that may damage the epithelial cells in the upper airways through peroxidation of cell membranes constituents and induces inflammatory gene activation leading to the secretion of pro-inflammatory cytokines, resulting in inflammation [37]. The higher odds of elevated CRP among women has also been reported in other studies [38,39]. In contrast, Rifai et al. [40] reported comparable frequency distribution of CRP among apparently healthy men and women in the United States. This was however among individuals within the age range of 40 to 84 years. The discrepancy in CRP between men and women has been attributed, at least in part, to the use of hormone therapy, which has been associated with increased CRP concentration irrespective of type [41]. The study by Rifai et al. [40] excluded women taking hormone replacement therapy, thus it was not surprising that they reported comparable CRP distribution between men and women. In the current study, after female hormone use was adjusted for, females still had higher odds for elevated CRP concentration compared to males. This indicates that the higher odds of elevated CRP among women may not be due to hormone replacement therapy alone. Previous studies [39,42] have also identified an association between race/ethnicity and CRP concentration, with CRP lower among whites compared to non-white subjects. In this manuscript, although non-Hispanic blacks showed higher odds of elevated CRP concentration compared to non-Hispanic whites in the simple logistic regression (*p* = 0.001), this was not statistically significant in the multiple logistic regression (*p* = 0.06). In addition, while Ma et al. [43] have reported a significant association between fiber intake and CRP concentration, dietary fiber intake was not significantly associated with odds of elevated CRP after phytate intake and other factors were adjusted for in the current study. It is worth mentioning that foods high in fiber are also mostly high in phytate, and the inclusion of both dietary factors in the model might be the reason why fiber was not significant. This was done, however, to show that the relationship between phytate and CRP was independent of fiber intake. 

The findings of this study have important clinical and public health implications. It is estimated that nearly half of all adults in the United States suffer from one or more chronic disease [44]. Inflammation plays an important role in the development of several of these chronic diseases [14,15,16,17]. Dietary modification to include high phytate foods such as nuts, legumes and whole grains may play an important role in efforts to address inflammation and the concomitant adverse health outcome [45,46,47]. Among the limitations for this study are the fact that it is a cross-sectional study, implying that a causal inference cannot be drawn between phytate intake and CRP levels. Additionally, it cannot be determined from this cross-sectional study if prolonged consumption of high phytate foods is sufficient to maintain low inflammation and oxidative stress in obesity, thus warranting future longitudinal studies. Moreover, phytate intake data was estimated using data from another source apart from the NHANES data, although the source is internationally well recognized. In addition, the impact of food preparation on phytate intake was not accounted for in this study. It is also worth mentioning that the findings of this study only apply to individuals with overweight or obesity and not to those with normal or underweight.

## 5. Conclusions

The findings from this study show that intake of phytate is associated with reduced odds of elevated CRP concentration and highlight the importance of phytate in the prevention of inflammation-related chronic diseases. 

## Figures and Tables

**Table 1:** Background information of study participants 20 years and older with overweight or obesity as per the National Health and Nutrition Examination Survey (NHANES) of 2009/2010.

	*N* (Unadjusted)	Percentage (Adjusted) ^1^
Total	3152	100
Sex		
Men	1661	55
Women	1491	45
Ethnicity ^2^		
Non-Hispanic whites	1470	68
Non-Hispanic blacks	554	11
Mexican–Americans	674	10
Smoking status		
Never	1769	57
Current smoker	505	15
Former smoker	878	28
Medical Condition History ^3^		
None	1808	62
At least one	1344	38
Female hormone use		
Never used	2917	92
Current user	29	1
Former user	206	7

^1^ Adjusted for sampling weight, strata and primary sampling unit; ^2^ estimates for “other” race were not reported, although data were included in the overall analyses; ^3^ medical conditions included arthritis, congestive heart failure, coronary heart disease, angina/angina pectoris, heart attack, stroke, cancer, and diabetes.

**Table 2:** Phytate intake and C-reactive protein (CRP) concentration among subjects ≥20 years old with overweight or obesity as per the NHANES of 2009/2010 ^1^.

	*N*	Phytate Intake (g/d)	CRP (mg/L)
Total	3152	0.66 (0.64, 0.68)	1.4 (1.2, 1.5)
Sex			
Men	1661	0.74 (0.72, 0.76) ^a^	1.2 (1.1, 1.3) ^a^
Women	1491	0.59 (0.58, 0.61) ^b^	1.6 (1.4, 1.8) ^b^
Ethnicity			
Non-Hispanic Whites	1470	0.69 (0.66, 0.71) ^a^	1.3 (1.2, 1.5)
Non-Hispanic Blacks	554	0.53 (0.48, 0.58) ^b^	1.8 (1.6, 2.0)
Mexican Americans	674	0.67 (0.62, 0.70) ^b^	1.6 (1.5, 1.8)
Smoking status ^2^			
Never	1769	0.68 (0.65, 0.702) ^a^	1.3 (1.2, 1.4)
Current smoker	505	0.56 (0.51, 0.61) ^b^	1.5 (1.4, 1.7)
Former smoker	878	0.69 (0.67, 0.70) ^a^	1.5 (1.4, 1.8)
Medical Condition History ^2^			
None	1808	0.66 (0.63, 0.68)	1.2 (1.1, 1.4) ^a^
At least one	1344	0.68 (0.64, 0.70)	1.7 (1.5, 1.8) ^b^
Female hormone use			
Never used	2917	0.66 (0.64, 0.68)	1.3 (1.2, 1.4) ^a^
Current user	29	0.91 (0.58, 1.04)	1.6 (1.1, 4.1) ^a, b^
Former user	206	0.63 (0.53, 0.68)	1.9 (1.7, 2.3) ^b^

^1^ Values are medians with 95% CIs. Values with dissimilar alphabetical superscripts differ significantly (*p* ≤ 0.05, Bonferroni correction); ^2^ Medical conditions included arthritis, congestive heart failure, coronary heart disease, angina/angina pectoris, heart attack, stroke, cancer, and diabetes.

**Table 3:** Associations between phytate intake, demographic factors and odds of elevated C-reactive protein (CRP) concentration among subjects ≥20 years old with overweight or obesity as per the NHANES of 2009/2010.

	*N* (Unadjusted)	CRP ≤ 3mg/L	CRP > 3 mg/L	Odds Ratio (95% CI)	95% CI	*p*-Value ^1^
Phytate intake, g/day	3152	----	---	0.69	0.59, 0.81	0.0005
Fiber intake, g/day	3152	----	---	0.98	0.97, 0.98	<0.0001
Age, y	3152	----	---	1.00	1.00, 1.00	0.2
Sex						
Men	1661	77	23	RG		
Women	1491	58	42	2.4	2.0, 2.9	<0.0001
Ethnicity						
Non-Hispanic Whites	1470	69	31	RG		
Non-Hispanic Blacks	554	58	42	1.58	1.28, 1.96	0.001
Mexican Americans	674	70	30	0.94	0.74, 1.18	0.6
Smoking status						
Never	1769	70	30	RG		
Current smoker	505	65	35	1.27	1.04, 1.55	0.03
Former smoker	878	66	34	1.23	1.01, 1.49	0.06
Medical Condition History ^2^						
None	1808	70	30	RG		
At least one	1344	65	35	1.24	1.00, 1.54	0.07
Female hormone use						
Never used	2917	70	30	RG		
Current user	29	56	44	1.81	0.60, 5.45	0.3
Former user	206	52	48	2.09	1.59, 2.76	0.0001

RG, Reference group; ^1^ *p*-values are based on simple logistic regression analysis; ^2^ medical conditions included were arthritis, congestive heart failure, coronary heart disease, angina/angina pectoris, heart attack, stroke, cancer, and diabetes.

**Table 4:** Predictors of elevated CRP levels (CRP >3 mg/L) among subjects ≥20 years with overweight or obesity as per the NHANES of 2009/2010.

	Odds Ratio (95% CI)	95% CI	*p*-Value ^1^
Phytate intake, g/day	0.66	0.52, 0.84	0.04
Fiber intake g/day	1.00	0.99, 1.01	0.9
Age, y	1.00	0.99, 1.00	0.1
Sex			
Men	RG		
Women	2.20	1.84, 2.63	0.003
Ethnicity			
Non-Hispanic Whites	RG		
Non-Hispanic Blacks	1.39	1.12, 1.73	0.06
Mexican–Americans	0.98	0.77, 1.25	0.9
Smoking status ^2^			
Never	RG		
Current smoker	1.35	1.12, 1.62	0.05
Former smoker	1.40	1.16, 1.69	0.04
Medical Condition History ^2^			
None	RG		
At least one	1.14	0.90, 1.44	0.4
Female hormone use			
Never used	RG		
Current user	1.20	0.42, 3.47	0.8
Former user	1.31	0.94, 1.83	0.2

RG, Reference group; ^1^ *p*-values are based on a multiple logistic regression; ^2^ medical conditions included arthritis, congestive heart failure, coronary heart disease, angina/angina pectoris, heart attack, stroke, cancer, and diabetes.
